# Case Report: Ventriculoperitoneal Shunting and Radiation Therapy Treatment in a Cat With a Suspected Choroid Plexus Tumor and Hypertensive Hydrocephalus

**DOI:** 10.3389/fvets.2022.828083

**Published:** 2022-03-23

**Authors:** Elizabeth Mahon, Aldara Eiras-Diaz, Sarah Mason, Fabio Stabile, Ane Uriarte

**Affiliations:** ^1^Department of Neurology and Neurosurgery, Southfields Veterinary Specialists, Essex, United Kingdom; ^2^Department of Internal Medicine, Southfields Veterinary Specialists, Essex, United Kingdom; ^3^Department of Oncology/Radiation Therapy, Southfields Veterinary Specialists, Essex, United Kingdom

**Keywords:** ventriculoperitoneal shunt, hydrocephalus, choroid plexus tumor, radiation therapy, cat

## Abstract

A 14-year-old male neutered domestic short-hair cat was presented for a history of behavioral changes and episodes of urinary retention. Neurological examination was consistent with a multifocal intracranial neuroanatomical localization, with suspected right sided lateralisation and suspected raised intracranial pressure (ICP). Brain magnetic resonance imaging (MRI) revealed an intraventricular multilobulated well-defined T2W-hyperintense and T1W-isointense, markedly contrast enhancing mass lesion within the dorsal aspect of the III ventricle extending into the left lateral ventricle, causing hypertensive obstructive hydrocephalus. A ventriculoperitoneal shunt (VPS) was placed within the left lateral ventricle, followed by a radiation therapy (RT) course of 45 Gy total dose in 18 daily fractions. Six-months post-RT, computed tomography revealed mild reduction in mass size and resolution of the hydrocephalus. The patient was neurologically normal with no medical treatment. Raised ICP causes severe clinical signs, can lead to brain ischaemia and herniation, and significantly increases anesthetic risk during RT. Placement of a VPS in cats with hypertensive obstructive hydrocephalus may allow improvement of neurological signs due to raised ICP, and therefore making the patient a more stable candidate for anesthesia and radiation therapy.

## Introduction

Radiation therapy (RT) is an established treatment choice for inoperable intracranial tumors in cats (1). Ventriculoperitoneal shunt (VPS) placement has been described for the treatment of obstructive hypertensive hydrocephalus secondary to intraventricular tumors (2–4). To the best of the authors' knowledge, this is the first report of successful VPS placement and subsequent RT treatment in a cat with III ventricle tumor causing hypertensive obstructive hydrocephalus.

## Case Presentation

A 14-year-old, male neutered, domestic short hair cat presented after a 2-month history of episodes of urinary retention and behavioral changes. Before referral, the cat had been catheterised by the referring veterinary surgeon on several occasions after presenting with a distended, not easily expressible bladder. In between these episodes, it was reported that the cat would urinate once every 24 h and no periuria, stranguria or pollakiuria was noticed. There had been a gradual change in behavior, being more affectionate and disorientated at times. The disorientation increased and progressed to colliding into stationary objects the day before referral following sedation for abdominal imaging. Pre-referral, the following medications had been trialed: meloxicam (0.05 mg/kg q24 h PO) given for several days consecutively at various times in the two-month period when clinical signs began, prazosin (0.17 mg/kg q8–12 h PO) given for 7 weeks, two courses of amoxicillin-clavulanic (12.5 mg/kg q12 h PO) for a week each time and dantrolene (0.5 mg/kg q12 h PO). None of these had resulted in resolution of his urinary signs or altered behavior

On clinical exam, vital parameters were within normal limits. The body condition score was 5/9. On cardiac auscultation there was a grade 4/6 left apical systolic heart murmur. No abnormalities were detected on abdominal palpation. Neurological exam revealed mild obtundation and compulsive pacing with a tendency to circle to the right. There were inconsistent proprioceptive deficits mainly on the left side of the body. The menace response and oculocephalic reflexes were reduced bilaterally, and the pupillary light reflexes absent bilaterally. The neurological examination was consistent with multifocal intracranial neuroanatomical localization, with suspected right sided lateralisation. The main differential diagnoses at this stage were neoplasia or inflammatory granuloma.

Systolic blood pressure measurement was 130 mmHg. Serum biochemistry revealed raised creatinine kinase (2,241 U/L, reference range 50–200 U/L), alanine transaminase (111 IU/L, reference range 0–40 IU/L) and alkaline phosphatase (33 U/L, reference range 0–25 U/L). Hematology was unremarkable. Abdominal ultrasonography did not reveal any abnormalities and retrograde urethrocystogram did not show any obvious filling defects of the urethral lumen or urinary bladder. Urinalysis of a sample obtained by cystocentesis revealed a urine specific gravity of 1.042 (reference >1.035), a urine protein: creatine ratio of 2 (reference 0–0.2) and on dipstick evaluation a pH of 6, protein 3+ and hemoglobin-RBC 4+. The urine sample was grossly haematuric. Urine sediment analysis showed the presence of >100 red blood cells (reference <5/hpf) but was otherwise unremarkable. There was no growth on urine bacterial culture. Although glomerular disease could not be completely excluded, given the presence of gross haematuria, together with the absence of hypoalbuminemia, hypercholesterolemia or azotaemia, the increased urine protein:creatine ratio was considered most likely secondary to blood contamination due to the cystocentesis technique (5, 6).

Three-view, thoracic radiographs showed mild cardiomegaly with no radiographic evidence of congestive heart failure. Echocardiography was declined by the owner. Magnetic resonance imaging (MRI) was acquired from a 1.5-Tesla Philips Achieva Magnetic Resonance Imaging Scanner (Phillips Medical System, Eindhoven, Netherlands). Dorsal, transverse and sagittal T2-weighted images, dorsal, transverse and sagittal T1-weighted images plus T1-weighted images with Gadolinium contrast media, fluid-attenuated inversion recovery transverse images and T2^*^-weighted transverse images of the brain were obtained. MRI revealed a multilobulated, well defined T2-weighted hyperintense and T1 weighted isointense mass measuring 1.7 × 1.3 × 1.5 cm within the dorsal aspect of the III ventricle. There was marked heterogenous contrast enhancement. The mass extended within the left lateral ventricle and compressed the rostral aspect of the cerebellum caudally, the inter-thalamic adhesion rostro-ventrally and the pons ventrally. The lateral ventricles and olfactory recess were severely distended with normal signal intensity and sulcal effacement was noticed through the forebrain (Supplementary Figure 1). The MRI was consistent with an extra-axial mass within the III ventricle causing obstructive hypertensive hydrocephalus. The main differentials were a choroid plexus carcinoma/papilloma, ependymoma, subependymoma, meningioma, neurocytoma, pilocytic astrocytoma and oligodendroglioma. The collection of a cerebrospinal fluid (CSF) sample was not performed as it is contraindicated in cases of raised intracranial pressure as it increases the risk of brain herniation (7).

Treatment with 0.1 mg/kg of dexamethasone intravenously and 1 g/kg of mannitol intravenously was commenced followed 24 h later by 1.1 mg/kg of prednisolone PO q24 h and prazosin at 0.17 mg/kg PO q8 h which was continued until surgery 5 days later. At admission for surgery, the demeanor was slightly brighter, but the neurological exam was similar to presentation. No more episodes of urinary retention were observed. Under general anesthesia, a lateral approach to the parietal bone was performed. The temporal muscle was retracted, and a small, circular craniotomy was performed. The shunt was placed within the left lateral ventricle to the abdominal peritoneum (miniNAV® SHUNTSYSTEM with pediatric burrhole reservoir and differential pressure unit of 10 cm H_2_0). The craniotomy was sutured routinely without complications. Under the same anesthesia, a computerized tomography (CT) (Siemens Somatom Spirit) at 2 mm slice thickness and 1 mm collimation confirmed successful placement of the VPS and radiation therapy planning was performed. Intraoperatively, 20 mg/kg IV cefuroxime was given every 90 mins. After the procedure and overnight, treatment included 2 ml/kg/h of Hartmann's solution, 1 mcg/kg/ medetomidine as a constant rate infusion and 0.1 mg/kg methadone according to pain score using the Glasgow Feline Composite Measure Pain Scale.

The day after VPS placement, neurological exam had improved. There was still a tendency to circle to the right. The menace response and proprioception were normal and there were bilaterally reduced pupillary light reflexes. Twenty-four hours after surgery an episode of opisthotonos and ear twitching was observed. Seizure activity was suspected and 22 mg/kg of levetiracetam q8 h PO was therefore started. Twelve days after VPS placement, RT began. The gross tumor volume (GTV) was contoured to include all contrast enhancing tissue (Eclipse version 15). The clinical target volume (CTV) was agreed by two boarded radiation oncologists and was contoured to include the VPS and the lateral ventricles. A planning target volume of 3 mm was applied in all dimensions (see Supplementary Table 1). A bespoke dental mold bite block was made and fixed to a rigid Perspex positioning bridge. Prescription isocentre was to the midpoint of the PTV. No shielding was used. No bolus was used. Port intervals were performed at regular intervals during treatment. 45 Gy total dose was delivered using 6 MV photons in 18 daily fractions (total length over 23 days). The plan comprised of three wedged beams at G0G90G20.

Post VPS placement, 1.1 mg/kg prednisolone q24 h PO was continued for 2 months before tapering over 10 days and discontinuing. Levetiracetam was continued for 6 months. Six months after RT, no more seizure activity or urinary issues were reported. The behavioral changes had gradually returned to normal. On clinical and neurological examination no abnormalities were detected. Follow-up CT of the head at this time revealed a stable disease (1.7 × 1.3 × 1.3 cm compared with 1.7 × 1.3 × 1.5 cm previously) (Supplementary Figure 2) (8). The cerebellar shape was rounded in sagittal reformat and there was no trans tentorial herniation identified. The lateral ventricles were only slightly distended. The CT confirmed resolution of the hypertensive hydrocephalus.

At the time of writing, 10 months after VPS placement, the cat was on no medication and still according to the owner, free of clinical signs.

## Discussion

Choroid plexus tumors are rare tumors in cats, and the reported clinical signs are variable including seizures, blindness and altered mental status (1, 9). Although not mentioned if the cats in these studies had increased ICP, in people and dogs choroid plexus tumors are widely associated with raised ICP (2, 10). Ventricular shunt placement has been shown to significantly improve clinical symptoms of human patients with increased ICP secondary to brain tumors (11). A retrospective case series describing four dogs with brain tumors affecting the III ventricle reported that clinical signs such as abnormal mental status, unlocalizable pain, decreased menace response and impaired vision improved after VPS placement, demonstrating that these clinical signs were likely due to the raised ICP rather than the brain tumors themselves (2). Furthermore, a retrospective study reporting 45 dogs treated with VPS due to hypertensive hydrocephalus found that decreases in ventricular volume and increases in brain parenchyma after VPS placement have been associated with improvement in one or more pre-operative clinical signs in dogs (12). The cat in this case report had an improvement in neurological examination and clinical signs post VPS placement, demonstrating that the raised ICP was likely causing the presenting clinical signs.

There have been no previous reports of urinary retention in cats associated with brain tumors and/or hypertensive hydrocephalus. The micturition process involves the storage and emptying phases of the bladder and is controlled by both the autonomic and somatic nervous system (13). Urinary retention can be caused by both neurogenic and non-neurogenic disease, with the latter due to anatomic urethral outflow obstruction (14). The thorough investigations into bladder function, including urine analysis and culture, abdominal ultrasound and retrograde urethrocystogram, did not reveal a non-neurogenic cause. In the absence of consistent clinical signs or response to appropriate therapy, feline lower urinary tract disease was considered unlikely. Therefore, neurological disease was presumed to be influencing the cat's urine retention, particularly as there was resolution of clinical signs post treatment of raised ICP.

The voiding of urine is coordinated by the micturition center and involves detrusor muscle contraction and urethral sphincter relaxation. Afferent impulses are transmitted to the sacral spinal cord when stretch receptors in the bladder wall are stimulated. These impulses ascend to the pontine reticular formation (the micturition center) and the cerebral cortex. Voluntary control of urination is the response of inhibitory influence from the cerebral cortex, basal nuclei, thalamus, hypothalamus and cerebellum on the micturition center (13, 15). In people, urinary tract retention has been described secondary to structural disorders in the frontal lobe, posterior fossa, hypothalamus, basal nuclei, paraventricular white matter, internal capsule, cerebellum, brainstem and thalamus (16–19). In cats, severe cluster seizures have also been associated with neurogenic urinary retention (20). In our case, the urinary signs resolved when the raised ICP was treated. It is thought that the urinary retention was caused by increased pressure on the intracranial structures involved in micturition, potentially the thalamus due to the location of the tumor. Lesions in the thalamus in people have been associated with urinary retention, but to the best of the authors knowledge, this has never been reported in veterinary medicine (18, 19).

Increased ICP can be caused by trauma, ischaemia or space occupying lesions such as neoplasia, cysts or inflammation. As ICP increases, cerebrovascular autoregulation is impaired causing a decrease in the cerebral perfusion and ischaemic damage. Additionally, there may be herniation of brain tissue and this ultimately can be life threatening (21, 22). The use of anesthetic agents can affect cerebral blood flow and further increase ICP, therefore increasing the chance of fatality (23). Multiple anesthetics are required for RT and so it is vital that increases in ICP are treated before commencing the course.

Medical management of increased ICP involves reducing CSF production through glucocorticoid and diuretics but this often only results in short-term improvement of signs. Electrolytes must be carefully monitored with the use of diuretics, particularly in combination with glucocorticoids, as electrolyte depletion is a common sequalae (21, 24). Frusemide, acetazolamide, mannitol and hypertonic saline have been described, with mannitol and hypertonic saline used in cases where it is required to rapidly reduce ICP (21, 25). Omeprazole has also been proposed as a possible treatment to decrease CSF production. It has been demonstrated in dogs and rabbits that CSF production decreases when treated with omeprazole by ventriculocisternal or intravenous administration (26, 27). However, a pilot study with 15 healthy beagles given oral omeprazole for 14 days suggested that CSF production was not affected by this medication (28). Further studies are therefore required to assess its effectiveness in reducing CSF production in cases of hydrocephalus, thus it was not used in this case. This cat received glucocorticoids and a dose of mannitol (1 g/kg IV) during general anesthesia when the images from the MRI demonstrated obstructive hypertensive hydrocephalus. This was sufficient to stop further deterioration before VPS placement.

Placement of a VPS is often the preferred option for hydrocephalus treatment, although this too comes with various complications such as mechanical shunt failure, infection, hemorrhage and over drainage (2, 21). In human medicine, rarely metastatic spread of neoplastic cells in the CSF through the VPS from the brain to the abdomen have been reported (29). Literature on VPS placement success and complication rate in cats is lacking. One study in human medicine suggested a complication rate of 23.8% in patients treated with a VPS secondary to both communicating and non-communicating hydrocephalus (30). A comparison between dogs with congenital hydrocephalus treated with VPS or medical management showed similar outcomes, although the median follow up time was only 9 months for medical and 15 months for surgical management (31). However, a case series with dogs with hydrocephalus secondary to tumors of the third ventricle found that the ICP measured considerably higher (28 mmHg and 31 mmHg) than the intracranial pressures found in dogs with communicating internal hydrocephalus (mean 8.8 mmHg, range from 3 to 18 mmHg) (2, 32). In these dogs with hydrocephalus secondary to III ventricle tumors, their clinical signs were not improved by medical management but significantly improved after being treated with VPS placement (2). This supports the use of VPS placement in obstructive hypertensive hydrocephalus patients particularly if not responding to initial medical management. In this case, the intracranial pressure of the cat was not measured, but the limited response to medical management in the interim between diagnosis and VPS placement was another indicator to proceed with the VPS placement.

Successful surgical removal of intracranial tumors has been well described in cats (1, 33). Nonetheless, for tumors in less accessible areas, radiation therapy is a viable alternative with one study finding that 95% of cats had an improvement in clinical signs after treatment (1). However, the lack of histological diagnosis for the tumor in this case and therefore the potential response to RT is a limitation of the case report. For tumors in the III ventricle, surgical resection has been successfully attempted in veterinary medicine (34, 35). However, due to the very challenging location, it carries a high rate of complications and post-operative mortality, hence was not attempted in this case (4, 36, 37). There has been one case report of successful surgical removal of a tumor from the III ventricle in a cat which was later diagnosed histologically as an ependymoma. In this particular case, RT was not available and so a left rostral tentorial craniotomy was performed and the III ventricle approached via the dilated left lateral ventricle (35).

Palliative VPS placement has been described in a cat with a later histopathological diagnosed ependymomas causing hypertensive obstructive hydrocephalus. However, the cat presented 6 months later with reoccurrence of neurological signs and was euthanised 10 months after VPS placement due to rapid deterioration (4). In dogs, both RT and VPS placement have been described for the treatment of ventricular tumors, but direct comparison of survival times is difficult due to variations in detailed reports of tumor location and clinical condition (2, 38). Our case presented a rapid improvement and 10 months after diagnosis, the cat was still neurologically normal and free of clinical signs.

This case report describes successful VPS placement in a cat with obstructive hypertensive hydrocephalus due to a mass lesion within the III ventricle. The cat's clinical signs improved after VPS placement and was therefore a more stable candidate for multiple general anesthetics for RT.

## Conclusion

VPS placement should be considered for cases of obstructive hypertensive hydrocephalus to improve clinical signs and the safety of multiple general anesthetics for RT. The combined treatment of VPS placement and RT makes for good long-term outcomes.

## Data Availability Statement

The original contributions presented in the study are included in the article/Supplementary Material, further inquiries can be directed to the corresponding author/s.

## Ethics Statement

Ethical review and approval was not required for the animal in this study because it is a retrospective case report. Written informed consent was obtained from the owners for the participation of their animal in this study.

## Author Contributions

EM: article writing. AU: main clinician during case presentation and performed the surgery. AE-D: performed internal medicine consultation. SM: radiation planning. FS: clinical support. All authors contributed to the article and approved the submitted version.

## Conflict of Interest

The authors declare that the research was conducted in the absence of any commercial or financial relationships that could be construed as a potential conflict of interest.

## Publisher's Note

All claims expressed in this article are solely those of the authors and do not necessarily represent those of their affiliated organizations, or those of the publisher, the editors and the reviewers. Any product that may be evaluated in this article, or claim that may be made by its manufacturer, is not guaranteed or endorsed by the publisher.
