# Supplementary material for: Case Report: Ventriculoperitoneal Shunting and Radiation Therapy Treatment in a Cat With a Suspected Choroid Plexus Tumor and Hypertensive Hydrocephalus
Source: Front Vet Sci. 2022 Mar 23;9:828083. doi: 10.3389/fvets.2022.828083 (PMC8989464; doi:10.3389/fvets.2022.828083)
Supplement: Supplementary file 1 [file Data_Sheet_1.docx]

Supplementary Material

## Supplementary Figures

a)
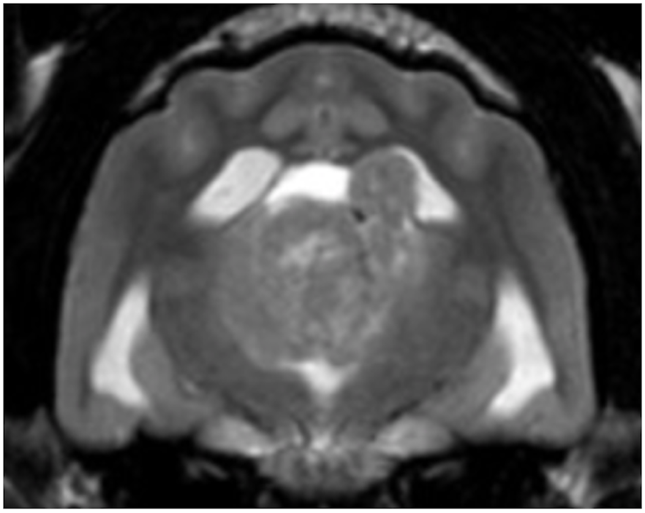


b)
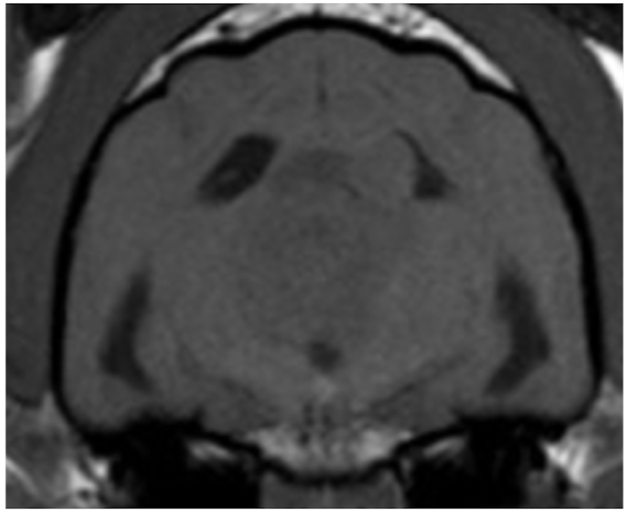


c)
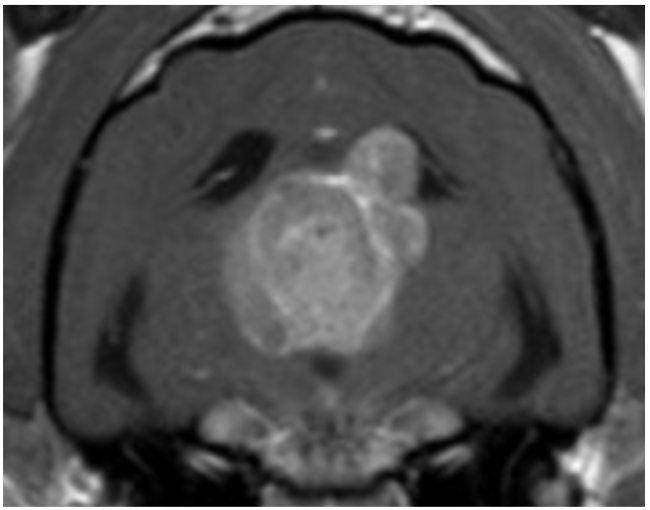


d)
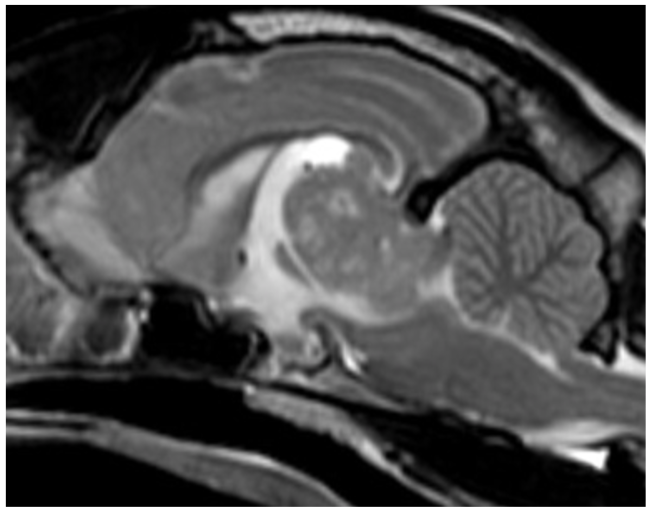


**Supplementary Figure 1:** T2-weighted (a), T1-weighted (b) and T1-weighted post gadolinium (c) magnetic resonance images (MRI) at the level of the thalamus, and T2-weighted midline sagittal MRI of the cat’s brain (d). The mass lesion (white arrow) measuring 1.7 x 1.3 x 1.5 cm within the dorsal aspect of the III ventricle extending within the left lateral ventricle and compressing the rostral aspect of the cerebellum caudally, the inter-thalamic adhesion rostro-ventrally and the pons ventrally. The lateral ventricles are severely distended with normal signal intensity and there is sulcal effacement throughout the forebrain.

1.
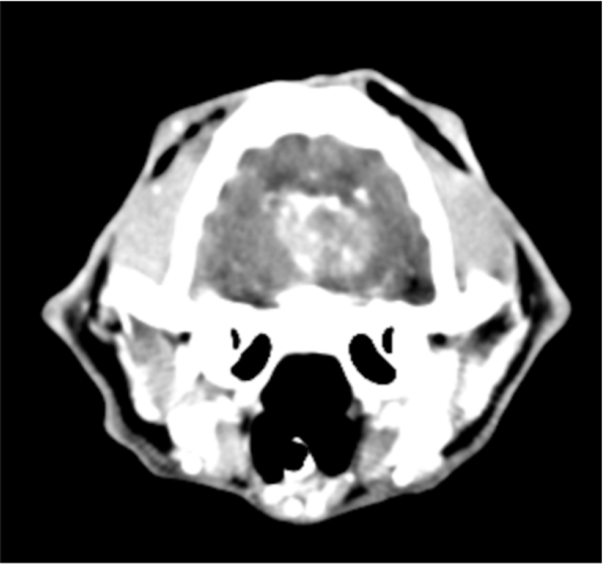

2.
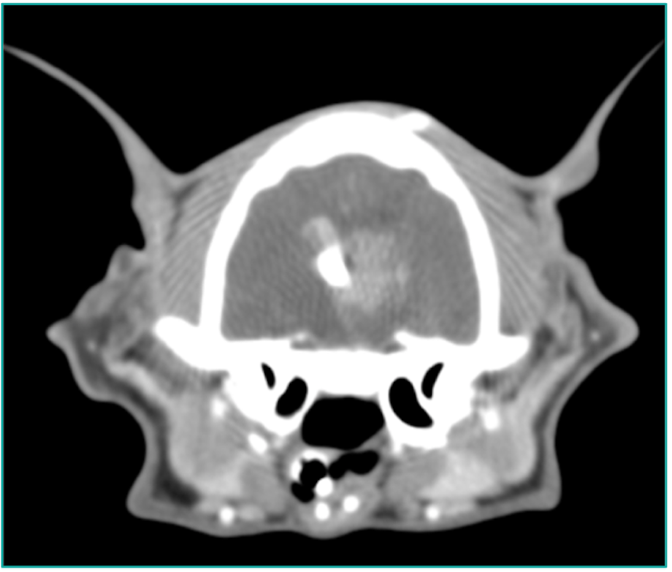


**Supplementary Figure 2:** CT immediately after VPS placement (a) and follow up CT 6 months later (b). Both images show the VPS (black arrow) and contrast enhancement of the mass (white arrow) measuring in a) 1.7cm x 1.3 x 1.5cm and b) 1.7 x 1.3 x 1.3 cm.

|  | Volume (cm3) | Minimum dose (%) | Maximum dose (%) | Mean dose (%) |
| --- | --- | --- | --- | --- |
| GTV | 2.1 | 98.8 | 100.7 | 99.8 |
| CTV | 10.6 | 97.5 | 102.3 | 99.6 |
| PTV | 21.7 | 91.5 | 102.3 | 99.2 |
| cerebrum | 25.3 | 98.9 | 102 | 98.2 |
| cerebellum | 4.3 | 32 | 101 | 90.3 |
| brainstem | 2.4 | 12 | 99 | 69 |

Table 1: Volume and doses of GTV (gross tumour volume), CTV (clinical target volume), PTV (planning target volume), cerebrum, cerebellum and brainstem.
